# Characterization of Microbial Communities in a Dairy Farm Matrix in Ningxia, China, by 16S rDNA Analysis

**DOI:** 10.1155/2019/3827360

**Published:** 2019-09-08

**Authors:** Wen Zhang, Wu Li, Chenjie Ma, Xiaoling Wu, Xunde Li, Jin Zeng, Guangcun Deng, Yujiong Wang

**Affiliations:** ^1^Key Laboratory of Ministry of Education for Conservation and Utilization of Special Biological Resources in the Western, China; ^2^College of Life Science, Ningxia University, Yinchuan, Ningxia 750021, China; ^3^Western Institute for Food Safety and Security, 1477 Drew Avenue, Suite 101, Davis, California 95616, USA; ^4^Department of Population Health and Reproduction, 1089 Veterinary Medicine Drive, Davis, California 95616, USA

## Abstract

A large amount of dairy manure is produced annually in the Ningxia Hui Autonomous Region of China due to the increase in food-producing animal agriculture in this region. The presence of bovine-originated zoonotic, especially human, pathogenic bacteria in untreated manure poses a significant threat to the environment and to public health. However, little is known about the composition, diversity, and abundance of bacterial communities in untreated dairy manure in the Ningxia region. In this study, the microbial community structure of the dairy farm matrix was characterized through 16S rDNA sequencing. The impact of manure treatment methods on bacterial communities was also analyzed. The results showed that the microbial community in dairy manure contained both beneficial bacteria and pathogens, with *Firmicutes*, *Bacteroidetes*, *Proteobacteria*, *Spirochaetes*, and *Actinobacteria* as dominant phyla. The results also showed the diversity and variety of abundance of zoonotic pathogens among different matrices. The number of pathogens was found to increase significantly in the accumulated but untreated manure, which appeared to be the main matrix of dairy farms that accumulated pathogens including zoonotic pathogens. Findings from this study suggested that farm management, particularly proper treatment of manure, is essential to achieve a shift in the bacterial community composition and a reduction in the environmental load of pathogens including zoonotic pathogens.

## 1. Introduction

The growth of the dairy industry has been accompanied by an increased volume of waste emissions that mainly consist of fecal and farm matrices. Manure contains a large number of undigested organic nutrients such as sugars, amino acids, nucleic acids, and vitamins. It is thus a valuable source of organic matter, nitrogen, phosphorus, potassium, and some micronutrients [1]. Animal manure has therefore been used on farms as one of the most important and valuable sources of nutrients to improve soil fertility and increase agricultural crop production. Some farms recycle the solids in manure to use as bedding material, which can have advantages for farmers in terms of availability, convenience, and cost-effectiveness [2]. Researches have demonstrated that the use of organic manure, whether it is used alone or in combination with inorganic fertilizers, can have positive effects on crop yield and can improve the soil quality [3, 4]. In China, manure has been used in many agricultural regions of the country for centuries. It is also widely used agriculturally in other parts of the world such as the UK and the USA.

The application of manure to agricultural land is an environmentally friendly method of waste disposal. However, in addition to organic matter, manure also contains many harmful gases, heavy metals, parasite eggs [5], antibiotic resistance genes [6, 7], and a variety of intestinal microflora and opportunistic pathogens, as well as antimicrobial-resistant bacteria [8]. Pathogenic and antimicrobial-resistant microorganisms contained in the manure can lead to the contamination of edible agricultural products [9, 10]. Thus, if these manures are used as fertilizer without treatment or are not treated properly, dangerous microorganisms could be transferred from animals to humans, bringing about a threat to the environment and to human health. In addition, bacterial contamination of dairy farm environments can cause disease or spoilage of milk and its secondary products [11]. The most harmful diseases are mastitis and foot rot [12], which reduce the quality of dairy products, inhibit the development of the dairy industry, and have a negative impact on food security and human health [13, 14]. Bovine mastitis is a major disease affecting the dairy industry worldwide with huge economic losses and decreased animal health. Furthermore, it is a common but complicated disease in high-yielding dairy farms. During the course of dairy cow breeding, it is quickly spread and difficult to cure, resulting in serious economic losses [15].

Farm management, particularly proper treatment of manure, has become an issue of concern in many farms. China has the largest population of livestock animals of any country in the world; however, many livestock farms have poor animal manure management facilities for the treatment and disposal of manure [16, 17]. It is therefore suggested that manure management needs to be improved in China [17]. An increasing number of studies have found that poor farm management can lead to severe environmental problems such as the pollution of air, water, and land [10, 18, 19]. The milk industry is one of the five leading strategic industries in the Ningxia region of China. However, few studies have examined the effect of poor dairy farm management on microbial community compositions and diversity among the dairy farm matrix in this region. By using pyrosequencing of metagenomic 16S rDNA, the objective of this study was to characterize bacterial diversity in feces, manure, and soils in dairy farms in the Ningxia region of China.

## 2. Methods and Materials

### 2.1. Sample Collection

Three dairy farms representative of typical dairy farm operations in the Ningxia region were enrolled in this study. Farms 1, 2, and 3 were located in the suburbs of Xingqing (XQ), Jinfeng (JF), and Xixia (XX) districts of Yinchuan City, respectively. Manure from these dairy farms has been used as organic fertilizer by local produce farmers. Between April and July of 2016, fresh feces, manure, and soil samples were collected from these farms. Fresh fecal (F) samples were collected within 30 seconds of excrement from lactating cows. Manure (M) samples were collected from piles of accumulated manure without further treatment. Soil (S) samples were collected from around the farms at different depths (0, 10, and 20 cm). To eliminate error caused by individual differences or unrelated factors, fresh fecal samples were a blend of at least six cow feces and manure and soil samples were a blend of at least six sampling sites or depths. Each sample from each farm was mixed separately, with a total of 27 samples being generated after mixing. All samples were refrigerated immediately after collection and during transportation to the laboratory. Upon arrival at the laboratory, samples were stored at −80°C until processing.

### 2.2. DNA Extraction and Sequencing by Synthesis

Metagenomic DNA was extracted from all types of samples using a QIAampR DNA Stool Mini Kit (Qiagen, Mississauga, Canada) according to the manufacturer's instructions. The extracted DNA was analyzed by electrophoresis on 1% agarose gels and then stored at −80°C before further analysis. PCR was performed using a Phusion High-Fidelity PCR Master Mix (New England Biolabs (Beijing) Ltd., China) under the following conditions: 94°C for 3 min (1 cycle), 94°C for 45 s, 50°C for 60 s, 72°C for 90 s (35 cycles), and 72°C for 10 min. PCR products were purified using the QIAquick Gel Extraction Kit (Qiagen, Dusseldorf, Germany). Briefly, the DNA was amplified by the primer set 515F and 806R, which targeted the V4 region of the bacterial 16S rDNA, with the reverse primer containing a 6 bp error-correcting barcode unique to each sample [20]. Sequencing by synthesis was performed on an Illumina HiSeq 250 platform (Novogene Bioinformatics Technology Co., Ltd., Beijing, China).

### 2.3. Bioinformatics and Statistical Analysis

Sequence analysis was performed using the Sparse software (Sparse v7.0.1001). Sequences with ≥97% similarity were assigned to the same operational taxonomic unit (OTU). The representative sequence for each OTU was screened for further annotation [21]. Sample reads were assembled using mothur v1.3213 [22]. Moreover, high-quality sequences were aligned against the SILVA database (version 115) [23]. Sequences were further qualitatively trimmed using a 2% cluster error [24, 25], and chimeras were removed using UCHIME [26]. Assignment of OTUs was performed at 97% identity using the furthest neighbor algorithm. Taxonomic assignments were made against the Ribosomal Database Project database (version 9) [27]. For comparisons, groups were normalized to include 27 samples, each randomly subsampled to 25,000 sequence reads (275,000 sequence reads per group). For determination of the percentages of sequence reads and OTUs (97% sequence similarity) unique to each group, no normalization was performed.

The UniFrac distances were calculated using QIIME software (version 1.7.0), and these data were used to build the UPGMA sample cluster tree. Jackknifed beta diversity included both unweighted and weighted UniFrac distances calculated with 10 times subsampling, and these distances were visualized by principal coordinate analysis (PCoA) [28]. Principal component analysis (PCA), PCoA, and nonmetric multidimensional scaling analysis (NMDS) graphs were drawn using the R software (version 2.15.3).

Taxonomy assignment of OTUs was performed by comparing sequences to the Greengenes database (gg_13_5_otus). The Mann–Whitney *U* test was used to test for the significance of alpha diversity. Two-sided Student's *t*-test was conducted to determine the significance of beta diversity between sample groups. Linear discriminant analysis coupled with effect size (LEfSe) was performed to identify the bacterial taxa represented between groups at the genus or higher taxonomy levels [29]. The functional profiles of microbial communities were predicted using PICRUSt [30]. The bootstrap Mann–Whitney *U* test with 1000 permutations was also used to identify gene pathways or OTUs with significantly different abundances between groups. The R packages “phyloseq” and “heat map” were used for data analysis and plotting [31, 32].

## 3. Results

### 3.1. Bacterial Community Composition in the Dairy Matrix

In total, 2.2 million strands of 16S rDNA amplicon data were generated from the 27 samples using pyrosequencing. After trimming and cleaning, this number was reduced to 69,065 high-quality reads with a median length of 253 bp. Only 1.5% of the sequences were identified as chimeras and were excluded from further analysis. The number of sequences in the 27 filtered samples was in the range of 47,924 to 83,624 sequences, and after homogenizing these sequences, the sequences were concentrated to around 45,000. An OTU table was generated by clustering all of the sequences into OTUs with a 97% similarity level. The species observed among the samples showed that the same samples from different farms possessed the same patterns, with the number of microbial notes in soil being higher than that in other samples. The samples were grouped by category, and the main annotations are shown in Table 1.

**Figure 1:**
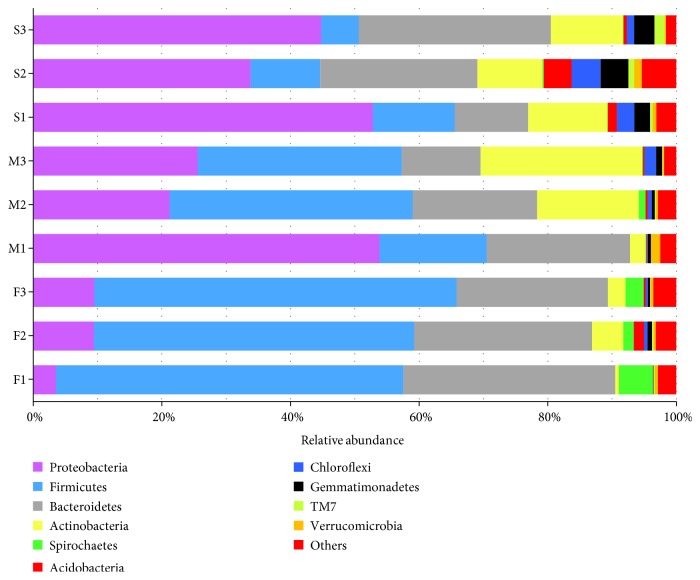
Bacterial community distribution in the F1, F2, F3, M1, M2, M3, S1, S2, and S3 samples at the phylum level (top 10 phyla). F: fresh feces; M: manure; S: soil; 1: dairy farm in XX district; 2: dairy farm in JF district; 3: dairy farm in XQ district.

**Figure 2:**
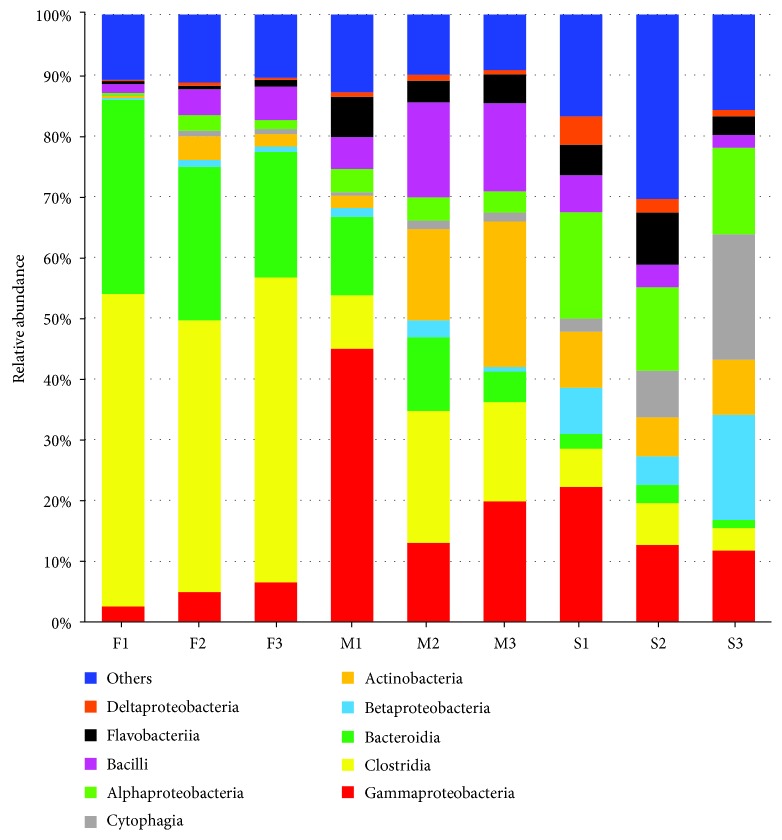
Bacterial community distribution in the F1, F2, F3, M1, M2, M3, S1, S2, and S3 samples at the class level (top 10 classes). Based on the results of annotation, the top 10 units with the highest abundance at the class level were selected for each sample or each group, and the relative abundance columnar cumulant map was generated to visualize each relative abundance of units and their proportions at the class level. F: fresh feces; M: manure; S: soil; 1: dairy farm in XX district; 2: dairy farm in JF district; 3: dairy farm in XQ district.

A bacterial community bar chart of all samples was constructed at the phylum level, from which 47 units were annotated from fecal samples, 44 units were annotated from manure samples, and 50 units were annotated from soil samples (Figure 1 includes only the top 10). The percentages of each of the top 10 phyla in all of the samples are shown in Table 1. These phyla were abundant and accounted for >94.63% of the entire bacterial communities in all of the samples. Therefore, these 10 phyla of bacteria were chosen for further analysis. In the class-level analysis, 75 classes of bacteria were detected among the 10 phyla (Figure 2). At the order level, 63 bacterial orders were noted from the *Proteobacteria.*

### 3.2. Dynamics of Bacterial Diversity

The distribution of the relative abundances of bacteria at the phylum level varied among fresh feces, manure, and soil samples. The dominating phylum of bacteria in fresh feces was *Firmicutes* (53.40%), followed by *Bacteroidetes* (28.01%), *Proteobacteria* (7.52%), and *Spirochaetes* (3.27%). In manure, the dominating phylum was *Proteobacteria* (33.56%), followed by *Firmicutes* (28.75%), *Bacteroidetes* (17.95%), and *Actinobacteria* (14.45%). In soil, the dominating phylum was *Proteobacteria* (43.79%), followed by *Bacteroidetes* (21.91%), *Actinobacteria* (11.19%), and *Firmicutes* (9.83%) (data not shown). Data show that *Proteobacteria*, *Bacteroidetes*, and *Firmicutes* were the main phyla in all types of samples, although the percentage of *Firmicutes* was slightly lower than that of *Actinobacteria* in the soil samples. The three phyla were distributed at approximately similar ratios in the two manure samples (M2 and M3), while *Proteobacteria* dominated in one manure sample (M1). In contrast to the fecal and manure samples, *Proteobacteria* dominated the bacterial communities in all of the soil samples (Figure 1).

In fresh feces, the phylum *Firmicutes* was predominantly composed of the three classes *Clostridia*, *Bacilli*, and *Erysipelotrichi*, the phylum *Proteobacteria* was comprised mainly of the classes *Gammaproteobacteria*, *Betaproteobacteria*, *Alphaproteobacteria*, *Deltaproteobacteria*, and *Epsilonproteobacteria*, the phylum *Bacteroidetes* was mainly composed of the classes *Bacteroidia*, *Cytophagia*, *Sphingobacteriia*, *Saprospirae*, *Rhodothermi*, and *Flavobacteria*, and the phylum *Spirochaetes* was mainly composed of the classes *Spirochaetes*, MVP-15, *Brevinematae*, and *Leptospirae* (Figure 2 and data not shown). In manure, the compositions of the classes in the phyla *Proteobacteria*, *Firmicutes*, and *Bacteroidetes* were similar to those in fresh feces. However, the phylum *Firmicutes* in manure consisted of an additional class called AHT28. In addition, the phylum *Actinobacteria* in manure was mainly composed of the classes *Actinobacteria*, *Acidimicrobiia*, *Thermoleophilia*, *Rubrobacteria*, *Nitriliruptoria*, and *Coriobacteriia* (Figure 2).

**Figure 3:**
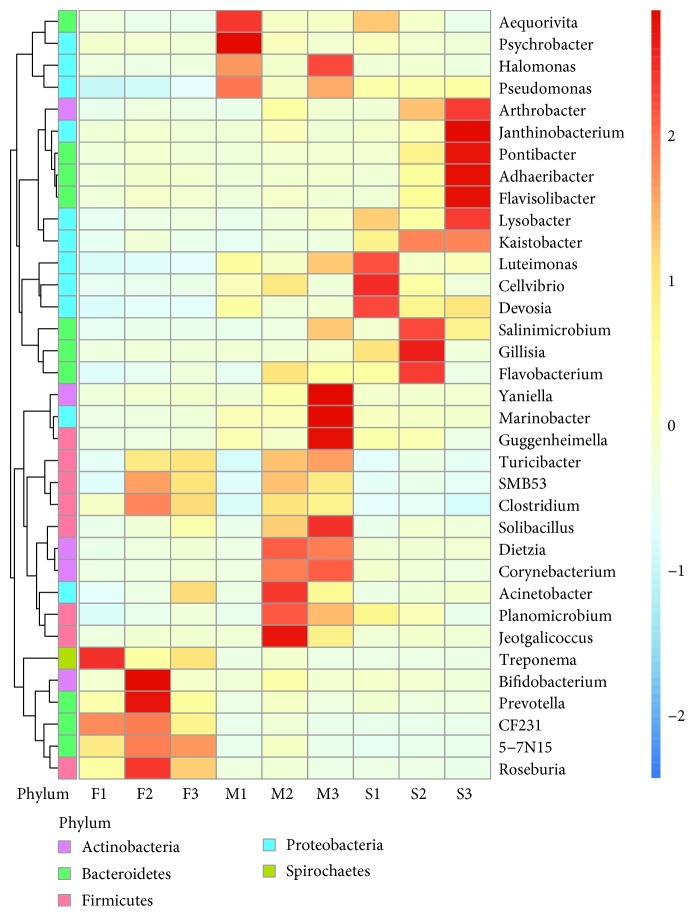
Heat map of the bacterial community composition of the F1, F2, F3, M1, M2, M3, S1, S2, and S3 samples. Heat map represents the natural log-transformed abundance of OTUs within the genus level in all of the different samples (fresh feces, manure, and soil) at the 97% nucleotide identity level. The corresponding values of the intermediate heat map are the *Z* values obtained by normalizing the relative abundance of each line of species; i.e., the *Z* value of a sample on a certain classification is the relative abundance of the sample on that classification and the relative abundance of all samples in that class. The difference in mean relative abundance is divided by the standard deviation of all the samples in that class. The vertical value is for the sample information, the horizontal value is for species annotation information, and the scale to the right indicates the phylum level of the microbial communities.

The data within the OTU table was compared between 5,000 randomly selected samples each at a 97% nucleotide identity level. To investigate variations in the distributions within microbial communities, all OTUs were assigned taxonomically using the RDP classifier. Among a total of 8819 OTUs, most bacteria were concentrated into five phyla, namely, *Firmicutes*, *Bacteroidetes*, *Proteobacteria*, *Spirochaetes*, and *Actinobacteria.* The heat map results showed that the compositions of the microbes in the same types of samples appeared to be similar (Figure 3).

**Figure 4:**
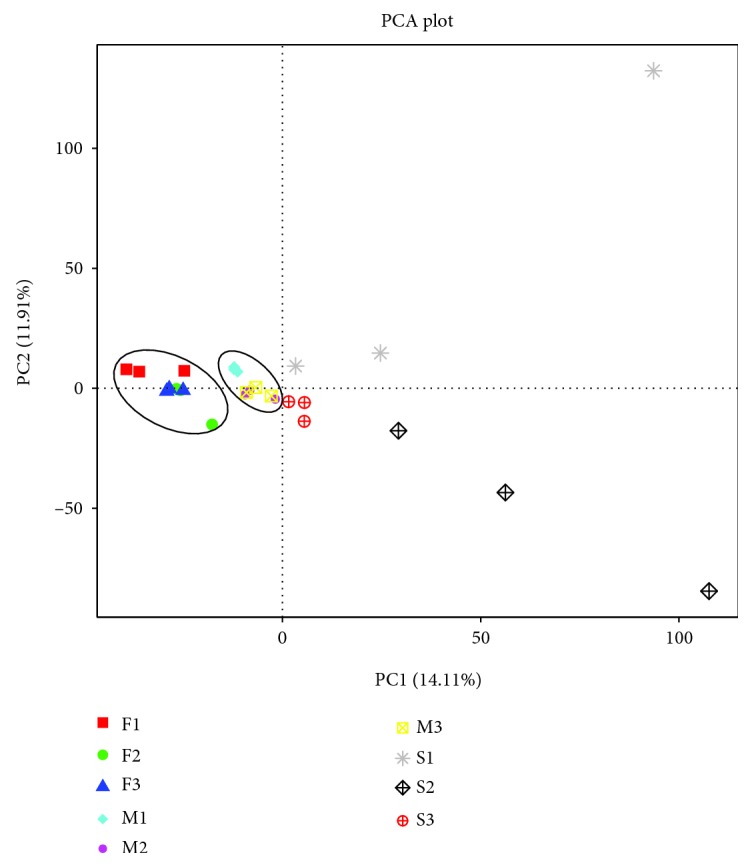
PCA between samples. F: fresh feces; M: manure; S: soil.

**Figure 5:**
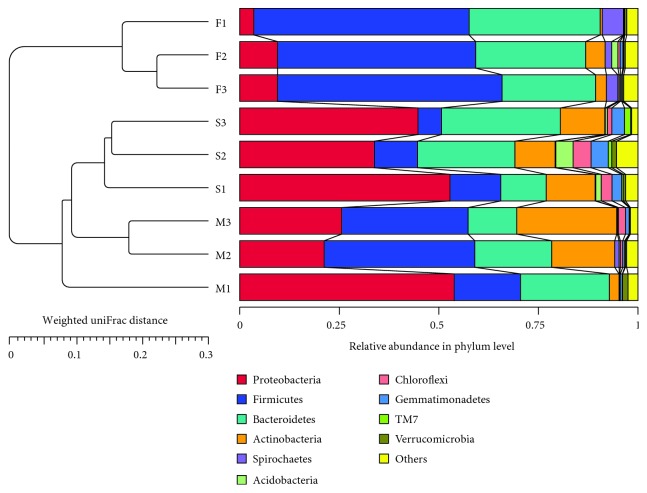
UPGMA clustering tree based on the unweighted UniFrac distances. On the left is the UPGMA cluster tree structure, and on the right is the relative abundance distribution of the units at the phylum level.

**Figure 6:**
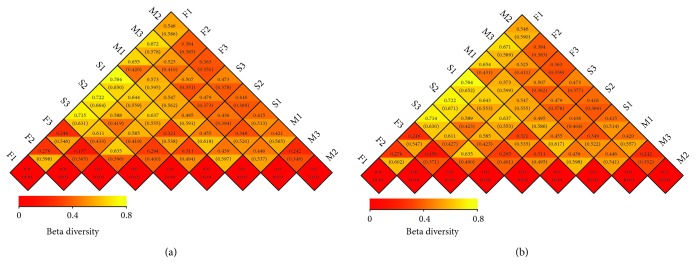
Beta diversity of general and HPB analysis among different samples. In the beta diversity study, the differences between the samples were measured by the weighted UniFrac distance and the unweighted UniFrac distance. The smaller the value, the smaller the differences in species diversity. In each diamond-shaped grid, the upper and lower values represent the weighted UniFrac distances and the difference between the samples, respectively. Graph (a) indicates bacterial diversity, and graph (b) indicates the diversity of human pathogenic bacteria.

**Figure 7:**
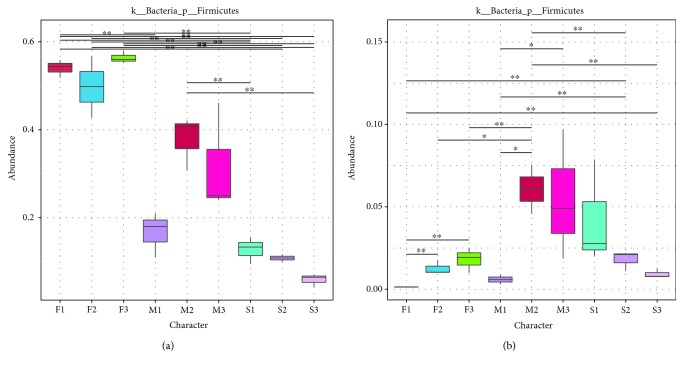
Box analysis of general and HPB analysis among different samples. In the figure, the horizontal axis shows the sample grouping; the longitudinal direction is the relative abundance of the corresponding species. The horizontal lines represent two groups with significant differences, while none indicates that there was no difference between the two groups. “∗” indicates a significant difference between the two groups (*q* value < 0.05), and “∗∗” indicates a significant difference between the two groups (*q* value < 0.01). The *p* value was obtained using the Met12aStat method to test the species abundance data between groups, and the *q* value was obtained by correcting the *p* value. Finally, the species with significant differences were selected according to the *q* values and the degree distribution box.

PCA profiles indicated that microbial communities varied depending on the type of sample. Principal components 1 and 2 (PC1 and PC2) demonstrated 14.11% and 11.91% of the total variance, respectively (Figure 4). PCA profiles showed significant separations between F1, F2, and F3 and M1, M2, and M3 treatments at three sites, especially for S1, S2, and S3. Treatments M1–M3 had higher scores than the corresponding treatments F1–F3 along the PC1 axis. According to the PC2 axis, PCA profile scores for treatment S3 were higher than those for both of the corresponding treatments S1 and S2. To determine the similarity between different samples, a clustering tree was constructed using UPGMA (unweighted pair-group method with arithmetic mean), which is a commonly used method for cluster analysis. Interestingly, the same type of samples from different farms clustered within the same branches (Figure 5). Further, beta analysis of microbial diversity showed that the diversity of species within the fecal, manure, and soil groups was rather small, in contrast to the large differences among the fecal and soil groups (Figure 6). In the analysis of human pathogenic bacteria, this trend was also detected. The boxplot showing the phylum level classification in terms of both bacterial diversity and the diversity of zoonotic pathogens revealed that the fresh feces contained a great abundance of *Firmicutes*, but a low diversity of zoonotic pathogens. By contrast, little change in diversity was observed in the accumulated manure or soil samples (Figure 7).

### 3.3. Diversity and Abundance of Zoonosis in Manure and Soil

**Figure 8:**
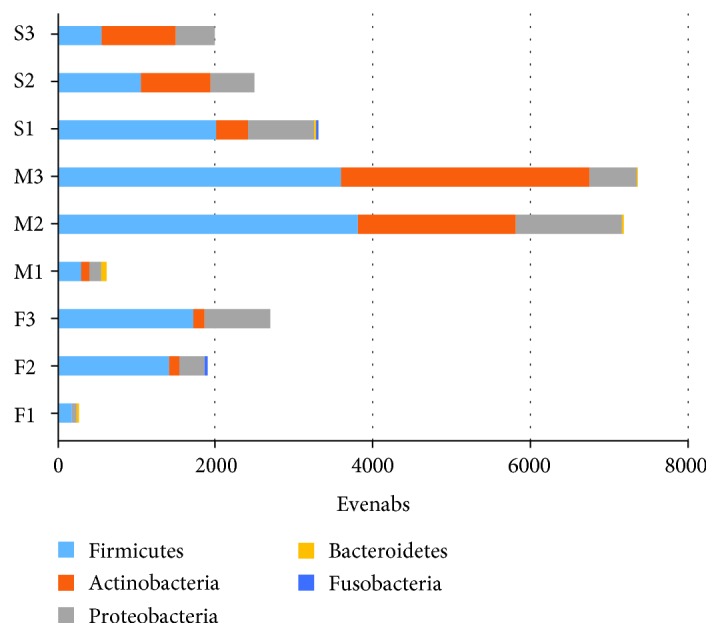
Bacterial community distribution of the F1, F2, F3, M1, M2, M3, S1, S2, and S3 samples at the phylum level for zoonotic organisms. F: fresh feces; M: manure; S: soil; 1: dairy farm in XX district; 2: dairy farm in JF district; 3: dairy farm in XQ district.

**Figure 9:**
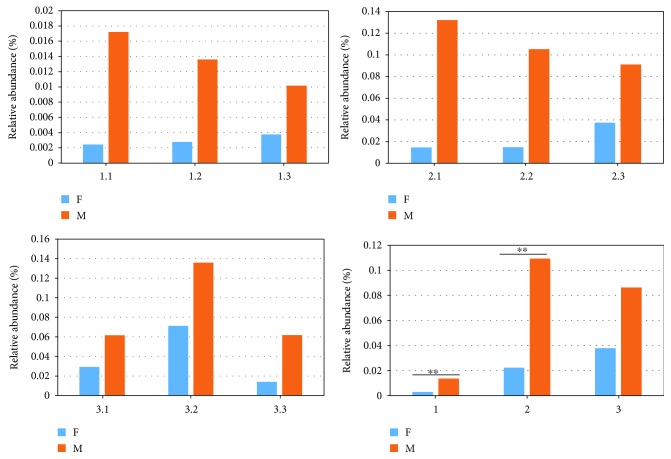
Statistical analysis of the zoonotic organisms (OTUs) at the order level of the F1, F2, F3, M1, M2, and M3 samples. F: fresh feces; M: manure; 1: dairy farm in XX district; 2: dairy farm in JF district; 3: dairy farm in XQ district.

The diversity and relative abundance of zoonotic pathogens in the manure and soil samples are shown in Figure 8. In total, 32 species of pathogenic bacteria were found in feces, manure, and soils. *Acinetobacter calcoaceticus* and *Bacillus cereus* were the dominant zoonotic species in feces, followed by *Enterococcus faecalis*, *Streptococcus uberis* 0140J, *Escherichia coli* O26: H11, *Corynebacterium diphtheria*, *Staphylococcus aureus* C0673, and *Pseudomonas aeruginosa*. The relative abundance of *B. cereus* in feces was 3 to 17 times higher, respectively, compared with that in manure and soil. Among the 32 pathogens, the relative abundance of 20 pathogens varied significantly in feces and manure, with the relative abundance in manure 2 to 19 times higher than that in feces. The number of *Actinomycetes* in manure was much higher than that in fresh feces. Further, at the genus level, although the number of genera in manure decreased, the number of bacteria and *Pseudomonas* increased significantly. This phenomenon was closely correlated with the abundances in feces and manure. In a more detailed classification order, the first dairy farm was detected having 27 zoonotic species, of which five species were increased in abundance in manure, including *S. aureus* M0406, *Clostridium perfringens B str.* ATCC 3626, *A. calcoaceticus*, *Bacteroides fragilis* NCTC 9343, and *Bacteroides vulgatus* CL09T03C04. In the second dairy farm, 34 zoonotic species were found, of which 14 species were increased in abundance in manure, namely, *B. cereus*, *Listeria monocytogenes FSL* R2-503, *S. aureus* M0406 and C0673, *E. faecalis*, *S. uberis* 0140J, *Streptococcus dysgalactiae*, *Clostridium botulinum*, *A. calcoaceticus*, *Acinetobacter baumanii BIDMC* 57, *Proteus mirabilis* BB2000, and *Vibrio cholera* VCC19. Finally, in the third dairy farm, 29 zoonotic species were found, of which 15 species showed increased abundance in manure, namely, *B. cereus*, *L. monocytogenes FSL* R2-503, *S. aureus* M0406 and C0673, *B. cereus*, *S. uberis*, *S. dysgalactiae*, *E. faecalis*, *P. aeruginosa*, *Klebsiella pneumoniae*, *C. diphtheria*, *Yersinia pestis biovar Antiqua* B42003004, *V. cholera* VCC19, and *B. fragilis* 3725-D9-ii. Statistical analysis of the OTU at the order level shows that the number of zoonotic bacteria in dairy farm 1 and dairy farm 2 was significantly higher (*p* < 0.01) in the manure than in the fresh fecal samples (Figure 9).

## 4. Discussion

In the Ningxia region, manure from most of the dairy farms is used as an organic fertilizer by local farmers without proper treatment. However, to our knowledge, the composition, diversity, and abundance of bacterial communities in the manure that has not been properly treated in this region are poorly understood, but this manure is being used as a fertilizer. Livestock and poultry manure contains feces, urine, litter, nose stains, blood stains, shed skin, hair, and placental material [8]. More than 150 species of microorganisms were identified in animal feces that can cause infectious diseases in humans including *E. coli*, *Salmonella*, *Giardia*, *Campylobacter*, and *Cryptosporidium parvum* [15, 33]. Some viruses in animal feces can also cause health problems to both livestock and humans. Researchers have found that *E. coli* O157: H7 can infect the edible part of lettuce through its roots [34]. Natvig and colleagues found that cleaning cannot effectively remove pathogens off the surface of vegetables [35]. Cow manure is frequently used as fertilizer that is spread onto the land for crop production [2]. Because of the presence of zoonotic pathogens in untreated manure, using such manure as fertilizer for crops may serve as a vehicle for pathogen transmission in the food supply chain.

In our study, the main phyla among the bacterial communities detected in the dairy farm matrix were *Proteobacteria*, *Firmicutes*, *Bacteroidetes*, and *Actinobacteria*, which have been reported to be common phyla of bacteria in both cow manure and soil [36, 37]. In addition to these common phyla, 16S rDNA sequence analysis also indicated that a large number of bacteria in the phylum TM7 were present in the samples analyzed in our study; in fact, it was the ninth most common phylum detected. TM7 has no known pure-culture representatives and is only characterized by 16S ribosomal DNA sequence data [38]. The phylum TM7 is widely distributed in the environment [39], and recent studies reported the existence of TM7 in the cattle gut [40, 41]. Although it remains to be confirmed whether TM7 in the cattle gut is part of the microbiota or an environmental contaminant, studies have demonstrated that TM7 is associated with human inflammatory mucosal diseases [42, 43] and oral disease [44]. The function of TM7 in the environment is still poorly understood due to the absence of pure cultures. The existence of TM7 in dairy fecal samples and manure samples in our study suggested that if this type of manure is used as fertilizer in farms, then caution should be taken since TM7 has been shown to be pathogenic.

The bacterial population in fresh feces is a mixture of the intestinal microorganisms, bacteria from the air, and digested foraged bacteria. Because fresh feces were collected immediately after defecation, the bacterial population in the fresh feces might be more similar to that in the intestinal environment. When compared with fresh feces, manure contained a less diverse bacterial community composition. This is probably due to the bacterial die-off that occurs naturally after fecal shedding and the accumulation of environmental pressures. In addition, the bacterial community structure changed during the process of manure accumulation due to bacterial interactions in this environment. Therefore, the bacterial population in manure consists of a mixture of soil bacteria and the newly established community. As reported by other researchers, the methods of manure treatment could alter the bacterial community in the manure, which was confirmed by analysis of the microbial population using sequencing methods [45]. Stacking of manure can result in alteration of the bacterial populations due to the exposure to oxygen that may typically promote the growth of facultative anaerobic organisms. Facultative anaerobes, which initially inhabited the intestines in relatively low numbers, may undergo anabiosis during the subsequent excretion process. We found that during feces accumulation, the types of microbial communities present declined, but the number of some of bacteria rose, which may be related to the proliferation of interstitial anaerobic bacteria. Our results demonstrated that the accumulation of manure leads to shifts in the bacterial community composition.

Our results indicated that approximately 50% of the bacterial population in feces comprised *Firmicutes*, which was consistent with previous reports. Most bacteria in the mammalian gut microflora are specific to the environmental niche of the gastrointestinal tract and are barely able to survive outside this environment [46]. This explains the decrease in gut bacteria in accumulated manure and the increase in other bacteria. Another cause for concern in manure is the presence of antibiotic resistance genes carried by certain pathogenic bacteria, such as those belonging to the family *Clostridiaceae*, including *C. tetani*, *C. botulinum*, and *C. perfringens* [47, 48]. These bacteria were also detected in the manure in our study. Moreover, the application of manure to soil impacts on the soil microbial community by introducing some bacterial taxa as well as new nutrients. The persistence of these bacteria in soil may enhance the likelihood of these bacteria entering the food chain through contaminated crops and becoming active antibiotic resistance gene donors when transferred back to anaerobic conditions in an animal or human gut [46].

*Acinetobacter*, *Pseudomonas*, and *Lysinibacillus* species are strictly aerobic bacteria and are not usual members of the gut microflora [49]. Although these species of bacteria may not be able to survive in the gut when facing different growth conditions and changed environments, they can potentially share and transmit antibiotic resistance genes or virulence genes to other bacteria. Other studies have corroborated the role of the *Acinetobacter* and *Pseudomonas* taxa in the persistence of antibiotic resistance genes in manure-treated soils [50]. In our study, we also found that three genera of bacteria appeared in all types of samples, with *Acinetobacter* and *Streptococcus* being more abundant in fresh feces and manure than in soil and *Pseudomonas* being more abundant in soil and manure than in fresh feces. The abundance of *Staphylococcus* in manure was higher than that in fresh feces and soil, the most important member of this genus being *S. aureus*. *S. aureus* is still described as one of the most frequently isolated etiological agents associated with bovine intramammary infections [51, 52], and some studies have shown a high incidence of *S. aureus* with genomic variation in resistance genes, which may pose a threat to public and animal health in Ningxia Province, China [53]. These pathogens displayed differences in antimicrobial resistance and could serve as carriers introducing antibiotic resistance genes into the food chain. Our results suggested that the application of poorly treated dairy manure as a soil amendment or organic fertilizer poses a potential risk for food safety and public health due to the potential transmission of antibiotic resistance genes or virulence genes and the possibility of introducing zoonotic pathogens into the environment. Therefore, accumulation of manure without any further treatment poses a direct hazard to the farm environment and the surroundings, which is a security risk for dairy farming and human public health.

Livestock manure is a source of pollution, but it is also a huge organic resource that can been used in many areas after proper treatment. There are many ways for treatment of livestock manure [54–56]. Decomposing components of livestock manure is a relatively common method. It is generally believed that composting at the temperature above 50°C for 5-10 days can meet the standard of harmlessness of manure [57, 58], but pathogenic microorganisms cannot be guaranteed during the accumulation process. The lower animals such as maggots and cockroaches can be used to decompose the manure of the livestock. This method can not only process livestock manure but also provide animal protein for feed. Maggots of the fly are very good animal protein feeds [59]. Biological fermentation is another way for treatment of livestock manure. After biological fermentation, harmful microorganisms such as pathogenic bacteria and parasitic eggs can be eliminated. Livestock manure has a high calorific value and can be used as a fuel to obtain heat [60, 61]. Livestock manure is rich in cellulose, which can also be used as a raw material to produce ethanol [62]. All of the above methods can eliminate pathogens to varying degrees; however, more research is needed to explain whether antibiotic residues in manure can be eliminated.

In summary, this study revealed the microbial community composition and diversity in the dairy farm matrix, as well as the abundance and distribution of pathogens. Our results demonstrated that accumulated manure that has not been subjected to further treatment may lead to a shift in the bacterial community composition and the enrichment of zoonoses. Dairy manure that has not undergone proper treatment therefore poses a threat to the environment and to public health. Therefore, developing and updating manure treatment practices should be considered a priority in dairy farm management. Our findings provide a theoretical basis for the necessity to treat dairy manure to prevent the spread of human pathogenic bacteria and other pathogens, thereby laying the foundation for sustainable local food-producing animal agriculture and protection of public health in the Ningxia region.

## Figures and Tables

**Table 1:** The phylum classification of the top 10 bacterial classes in each sample.

	F1 (%)	F2 (%)	F3 (%)	M1 (%)	M2 (%)	M3 (%)	S1 (%)	S2 (%)	S3 (%)
Proteobacteria	3.56	9.49	9.51	53.86	21.21	25.60	52.81	33.80	44.77
Firmicutes	54.04	49.77	56.38	16.66	37.82	31.75	12.75	10.85	5.90
Bacteroidetes	32.93	27.62	23.47	22.30	19.32	12.22	11.41	24.46	29.85
Actinobacteria	0.52	4.90	2.73	2.44	15.82	25.11	12.29	10.10	11.18
Spirochaetes	5.32	1.63	2.86	0.10	1.08	0.15	0.15	0.18	0.10
Acidobacteria	0.03	1.55	0.31	0.07	0.33	0.28	1.38	4.32	0.52
Chloroflexi	0.04	0.58	0.32	0.15	0.61	1.78	2.72	4.51	1.12
Gemmatimonadetes	0.05	0.69	0.35	0.51	0.49	0.93	2.40	4.33	3.20
TM7	0.18	0.22	0.10	0.05	0.17	0.14	0.42	0.88	1.55
Verrucomicrobia	0.53	0.35	0.39	1.40	0.32	0.13	0.58	1.19	0.21

## Data Availability

The data used to support the findings of this study are available from the corresponding authors upon request.
